# A methodological protocol for conducting a scoping review of health research on/by/with Indigenous women in North America

**DOI:** 10.1186/s13643-022-02080-6

**Published:** 2022-10-28

**Authors:** Keira A. Loukes, Celeste Ferreira, Janice Cindy Gaudet, Tricia McGuire-Adams

**Affiliations:** 1 School of Outdoor Recreation, Parks, and Tourism, Lakehead University, Thunder Bay, Canada; 2 School of Human Kinetics, University of Ottawa, Ottawa, Canada; 3 Campus Saint-Jean, University of Alberta, Edmonton, Canada; 4 Faculty of Education, University of Ottawa, Ottawa, Canada

**Keywords:** Indigenous health and wellness, Indigenous women, Scoping review, Theoretical frameworks, Methodology

## Abstract

**Background:**

Indigenous women in North America experience multiple inequities in terms of health and well-being when compared to non-Indigenous women and Indigenous men. In an effort to understand these health disparities, there has been a surge of research in the field of Indigenous women’s health and well-being over the last 20 years. The objective of this study is to conduct a scoping review of the most current research in this field to determine which theoretical frameworks are being used to study which topics in Indigenous women’s health and well-being in North America.

**Methods:**

The scoping review protocol used was designed to follow an iterative six-step process as laid out by Arksey and O’Malley. Peer-reviewed, academic articles from the following databases were identified: Academic Search Complete, Native Health Database, Web of Science, Google Scholar, Bibliography of Native North America, Sociological Abstracts, Gender Watch, and Indigenous Peoples of North America. Two team members subsequently conducted two screens of titles and abstracts to include articles which focused exclusively on Indigenous women’s health and well-being published between 2011 and 2021. The literature considered focused on Indigenous women’s health and well-being and explicitly states their use of critical theoretical frameworks (e.g., Indigenous feminist, intersectionality, Indigenous resurgence, feminist, critical race) or community-based participatory research (CBPR). Data analysis will involve quantitative and qualitative descriptions.

**Discussion:**

The results of our scoping review (in progress) will map out the current field of Indigenous women’s health research. Our findings will highlight the theoretical frameworks operationalized in research on Indigenous women’s health, identify gaps therein, and provide a basis for understanding how these theoretical lenses shape questions, methodologies, analysis, and implications of academic research.

## Introduction

Indigenous women’s health has become an increasingly popular subject of research over the last 20 years with the broad goals of better understanding the complexities of health, ill-health, well-being, and potential solutions to inequities and disparities [1–3]. Alongside this surge of interest, there has been a concerted effort to push back on “damage-centered research” [4]. By doing so, the research engages Indigenous peoples as co-researchers and therefore centers their respective Indigenous methodologies, theories, epistemologies, and methods. Strengthening theoretical frameworks that are regenerative and beneficial to Indigenous peoples, more specifically, women, is important given that Indigenous women continue to live with vast health disparities in comparison to Indigenous men and non-Indigenous peoples [5–8]. Increasingly, many studies, including the two recent Canadian National Commissions & Inquiries [9, 10], provide stark evidence that the roots of these disparities are formed and informed by colonial policies. Vital to decolonial health and well-being research is Indigenous women’s understandings, ethics, leadership, and knowledge systems that uphold our well-being. This work is bolstered by the thinking of Black feminist scholars such as hooks [11] whose work clearly demonstrates that “[Euro-centric] culture has not yet transformed in ways to support and sustain female well-being” (p. 141). Therefore, it is increasingly important to take account of the disciplines, topics, and theoretical lenses that inform questions, methodologies, approaches, and interpretations of research centering Indigenous women’s health and wellness. With this knowing, we conducted this scoping review to examine and reflects on the theoretical frameworks used within Indigenous women’s health research.

The frameworks utilized in Indigenous health research highlight underlying research paradigms. For instance, much critique is given to the use of positivist research frameworks in Indigenous health. More recently, McGuire-Adams [12, 13] clarified that Indigenous health research is challenged by its uncritical use of the deficit lens, which propels a whiteness centered, settler colonial view. The settler colonial view, she argued, substantiates Indigenous peoples’ collective disappearance vis à vis ill-health and eventual death [12, 13]. This stance corresponds with the striking results of a study that shows Indigenous women have the highest rates of mortality in Canada [5, 10]. Taking a critical approach to Indigenous women’s health research is necessary to untangle tokenism and settler colonial and white supremacist discourses inadvertently operationalized within it. It is from this critical lens of Indigenous women’s health from which we conduct our review of the frameworks currently being used in this field of research. The results of this scoping review are currently in preparation and will critically analyze the broader research paradigms being touted in Indigenous women’s health. We will publish the results of the scoping review in a subsequent article and present the implications for researchers involved in Indigenous women’s health and beyond.

In this article, we describe our methodology that guided our scoping review. This process follows Thiessen et al.’s [14] scoping review methodology paper on Indigenous perspectives on health and wellness in Canada, published in this journal.

## Methods

### Research team and framework for conducting the research

We are a research team made up of *cis*-gendered Anishinaabe, Métis, German-Scottish-Anishinaabe, and Black-mixed feminist scholars. Our scholarly work, which includes our everyday living, is invested in the field of Indigenous health, resurgence, and well-being. Trained and supported in critical theory, Indigenous research methodologies, community-engagement, and community-based participatory research methodology (CBPR), we approached our scoping review from Indigenous and Black feminist ontologies and epistemologies. Such collaborative approaches emphasize relationality, sharing, reciprocity, heart-led research, and a belief that knowledge itself has agency. For example, our meetings began with checking in on one another, a conscious beginning to share what was on our hearts and minds. Once each woman felt supported, our discussions about the scoping review would begin. We further recognized that the themes of our sharing were often tied with our decolonial critiques and analysis. This not only created space to support one another but also kept our reflections about our research alive, as it primed us to continuously reassess our purpose and goals in light of new experiences and realizations. Indeed, in this way, we wanted to make sure that in learning from Indigenous women’s health and well-being research, we were also *practicing the ethics of care and love for one another* through a way of being in relation that was taught and embodied by the generations of women that came before us [15]. Ethics of love literature from women of color feminists [11, 16, 17] is positioned as a form of colonial resistance and “as the practice of freedom” [18]. Nixon [19] stated that ethical love is a pedagogy that reflects being in good relation with all Creation through kinship responsibilities and attentiveness. This knowing is a part of wisdom traditions that are making their way into literature with the help of Black and Indigenous feminist researchers in relation to our wisdom keepers. While our relational ethics could be perceived as prolonging the research project, our collective experiences have shown that sharing our tears, laughter, rage, stories, and teachings immensely deepened and strengthened our ability to effectively collaborate on this project and to think more critically while fostering a wholesome self-esteem. We highly encourage collaborative research that values, respects, and enacts relational ethics upheld in Indigenous women’s leadership and mentorship approaches (McGuire-Adams T, Gaudet JC, Ward J: The emotional labour of reconciliation: indigenous women creating kind spaces through the complexities and challenges of reconciliation in the academy, forthcoming), [20–22].

Not only did this relational methodology immensely improve our experience of this project, it also improved the research itself—we were able to pivot, change, and address concerns and new perspectives in ways that allowed the project to grow and adapt. It allowed the work itself to be relevant, for the knowledge itself to have sovereignty. Although we are sharing our first step, our scoping review methodology did not follow a linear timeline. Instead, through this emergent process, we were scoping the field, analyzing the data, and shaping an anti-oppressive research tool through critical lenses simultaneously. We must follow this curved, cyclical process in our field in order to speak back to whiteness and to carve a space for Indigenous women’s approaches to knowledge making. This is especially important in the process of “producing” knowledge—which is truly not a production at all, but more a process of integrating with gikendaasowin, an Anishinaabeg concept for theory and knowledge [13, 23]. McGuire [24] describes gikendaasowin as the concept that encompasses all aspects of Anishinaabeg wisdom as has been passed through generations. It connects all the elements of Indigenous knowledge systems and necessitates that one engages “in the hard work of decolonization in order to recover and learn from Anishinaabe-gikendaasowin”. This is how this research makes a path for anti-colonial research; allowing us to weave Indigenous knowledges, ways of being, and kinship responsibilities into the academy, whereas often we feel we must divorce them [22]. Throughout this work, another question emerged as we asked ourselves, how can research in the academy foster and strengthen an ethic of love and care as informed by the Indigenous women’s worldview that we live in? This question will continue to underpin our work as we conduct the remainder of our scoping review, literature analysis, and anti-oppressive research framework tool, and will inform our perspectives and conclusions.

### Scoping review—justification and definition

We chose to approach this project with a scoping review as opposed to a systematic review as our goals are to map out the theoretical lenses used in the field of Indigenous women’s health and well-being over the last decade, identify gaps in this research, synthesize the existing knowledge [25–27], and “assess and understand the extent of knowledge in an emerging field” [28]. A systematic review, which aims to collect empirical evidence to answer a specific question [27], was not appropriate for the questions we were/are asking. A scoping review was the most useful approach because the research in Indigenous women’s health is varied and complex and our questions are exploratory in nature. We aim to ultimately advance the field of Indigenous women’s health and well-being by first determining what work has been conducted in the field, and what kind of theoretical frameworks and analyses are currently being used.

### Study design

This scoping review follows an iterative six step process as laid out by Arksey and O’Malley [29]. We also followed the Preferred Reporting Items for Systematic Reviews and Meta-Analysis extension for Scoping Reviews (the PRISMA-ScR) as updated by Peters et al. [28]. The steps we took are summarized as follows:

1. Identify the research questions. Our scoping review asks the following questions:
  - Which theoretical lenses are used by researchers examining Indigenous women’s health and well-being in Canada?
  - In the realm of Indigenous women’s health and well-being, how is the theoretical lens and methodology framed, conceptualized, and used?
2. Identify relevant studies in published academic journals. As our research questions are directed at theoretical and methodological approaches of researchers, we limited our search to peer-reviewed articles in academic journals. Due to the language abilities of our team, we limited our search to articles written in English.
3. Select studies to be included in analysis
4. Chart data gathered from selected studies
5. Collate, summarize, and report results
6. Consult with the wider community of experts in the field to identify gaps and strengths in our analysis.

### Data sources and research strategy

The two lead authors of the paper (a PhD candidate and Master’s student) met with a librarian at the University of Ottawa who focuses on Indigenous research in order to seek initial guidance in the scoping review. She helped us to organize our search process and data by suggesting keywords and the most appropriate databases to capture broad research from a variety of disciplines. She also suggested the use of Zotero, a literature and citation management tool, as a way to organize the articles we found. The suggested search strategy was discussed and approved by the full research team in subsequent meetings. Between June 25 and July 15, 2021, we searched the following databases: Academic Search Complete, Native Health Database, Web of Science, Google Scholar, Bibliography of Native North America, Sociological Abstracts, Gender Watch, and Indigenous Peoples of North America. Originally, we searched from the years 2000–2021. In subsequent meetings with our team, however, we decided to limit our search to the past decade in order to focus our project on the most recent research. Below is a description of the terms used in our search strategy for each database.

1. Indigenous OR Aboriginal* OR Native* OR “American Indian*” OR “First Nations” OR Inuit OR Metis

AND

2) wom?n OR girl* OR female* OR gender*

AND

3) health* OR “mental health” OR well-being OR “well being” OR wellbeing OR wellness OR medic* OR “physical activity” OR nutrition OR nutrient* OR “quality of life” OR illness OR “quality of life”

AND

4) “North America” OR Canada OR America OR “United States” OR “USA”

We acknowledge that these search terms may be limiting as they do not include specific nations. We chose to keep the terminology within the scope of First Nations, Métis, and Inuit for this broader scoping review given the diversity of specific nations and that most Indigenous health research uses general terms such as Indigenous or Aboriginal peoples. We acknowledge the importance of future research that offers a deeper analysis for nation-specific identities. As we prepared our final analysis, we conducted one more search on November 15, 2021, to capture any papers that had been published over the last few months.

### Citation management

Our team used Zotero to keep track of our citations. We developed a shared folder and created subfolders according to the year published and theoretical framework used. As we continued to screen articles to use in our study, we added new folders. This allowed all team members to be able to view the progress of the project, monitor the articles we were using, and identify some that were missing based on our experience in health and wellness-centered research. After we established a robust system of article management, we began to screen articles to use in our analysis.

### Eligibility criteria

The following steps were taken with more focused eligibility criteria in each screen as we proceeded through the scoping analysis.

1. Records identified through database search

Eligibility criteria:

- Published between January 2000 and December 2021
- Research must be focused on health or wellness of Indigenous women in North America

The articles that were first identified were based on our search terms which screened titles, abstracts, and keywords, as well as our specified time frame. We included articles that focused on Indigenous women and health and were based in North America. We identified appropriate articles by reading titles, abstracts, and keywords. Originally, we based this screen on all articles written between 2000 and 2021. One team member took on the years 2000–2015, and the other took on 2016–2021 to account for the expected surge in recent years. We transferred articles that met our eligibility criteria into separate Zotero folders based on year. Sometimes, we were able to separate them further based on the theoretical lens and methodology used. In this initial article identification, we attempted to separate articles broadly into quantitative physical sciences, quantitative social sciences, general qualitative research, community-based participatory research, and critical theory lenses. If the research framework was not clear, the article was placed in an “unidentified theoretical lens” folder. This initial article identification stage was useful as it gave us a broad picture of the overall volume and research approaches in the field of Indigenous women’s health and wellness.

2) Screen one:

Eligibility criteria:

- Published between January 2000 and December 2021
- Research must be focused exclusively on Indigenous women in North America
- Research must be focused on health (physical, mental, emotional, spiritual) and/or wellness/well-being

**Fig 1:**
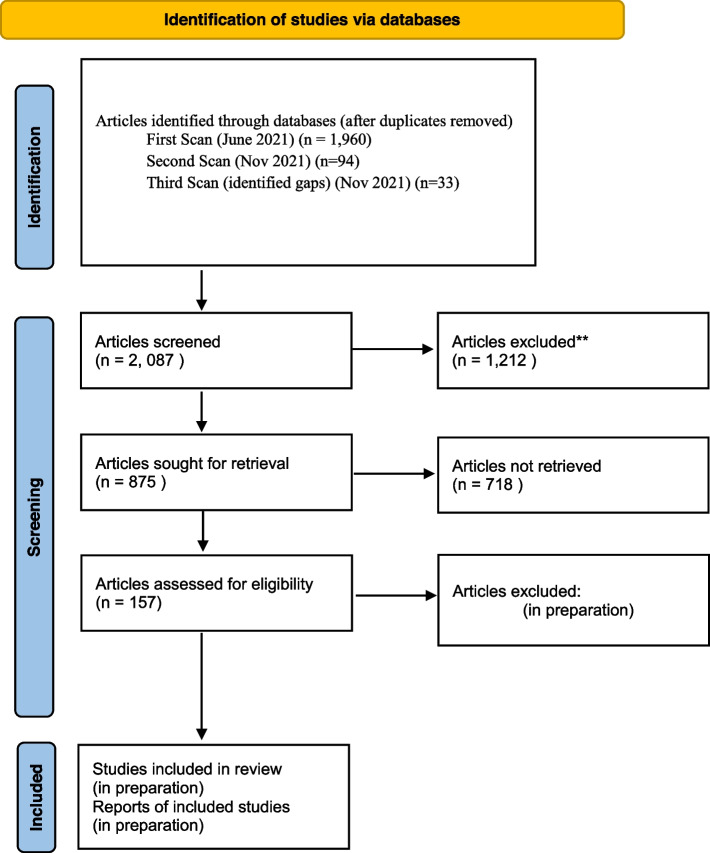
PRISMA flowchart detailing search retrieval and inclusion (*from:* Page MJ, McKenzie JE, Bossuyt PM, Boutron I, Hoffmann TC, Mulrow CD, et al. The PRISMA 2020 statement: an updated guideline for reporting systematic reviews. BMJ 2021;372:n71. doi: 10.1136/bmj.n71)

Due to the sheer volume of articles identified (see Fig. 1), we adapted eligibility criteria in order to focus on the most relevant articles. Since Zotero imports articles’ titles, authors, and abstracts, we were able to read through the titles and abstracts of each article a second time and excluded any article that did not solely focus on Indigenous women’s health and well-being. In those instances where the abstracts were not automatically imported, we used Google Scholar to search for the article and imported the metadata manually. In this first screen, we reviewed and further subdivided the articles into their respective folders based on the explicit theoretical lens the researchers used.

3) Screen two:

Eligibility criteria:

- Published between January 2011 and December 2021
- Research must be focused *exclusively* on Indigenous women in North America
- Research must be focused on health (physical, mental, spiritual) and/or wellness/well-being
- Research must *explicitly* state that it is using critical theory (e.g., feminist theory, Indigenous feminist theory, critical Indigenous theory, intersectionality theory, decolonial theory or community-based participatory research)

The results of our first screen left us with a list of articles too large to conduct an effective data extraction and analysis (see Fig. 1). We moved articles that met these criteria into respective theoretical lens folders in Zotero. On November 15, we conducted one more screen to capture any articles written in the past few months. After this, the third and fourth authors (the two principal investigators of the project) conducted a thorough review of the articles included to see if any important research in the field was left out. We subsequently conducted a third scan for eligible articles specific to these authors. This speaks to the importance of researcher engagement not only in the literature but also in the broader research community. Figure 1 presents a flow diagram of our screening results.

### Full text analysis, data extraction, and descriptors

For our final screening and analysis, we divided all the articles that made it through our screening between the team members. We created a shared Google Sheets folder with the following headings of information to be pulled from each article during the full-text analysis: author; title; abstract; journal; year; urban/rural; country; province; communities; participants; Indigenous authorship; female authorship; discipline; topic of study; research question; qualitative/quantitative/both; theoretical lens; methodology; methods; analysis; discusses intersecting forms of oppression (Y/N); discusses systemic racism (Y/N); discusses colonization as a determinant of health (Y/N); findings and recommendations; overall impressions (re: whether the article should be included or not supported by a rationale).

The methods used in the article analysis were piloted with a few articles in order to have consistency with the approach taken by the team. Adjustments were made accordingly. This analysis will determine which articles will be included in our final results.

### Data management

Data was and continues to be managed through a shared Google Sheets document in a shared folder that all members of the research team have access to. To avoid duplicating the work, each member of our research team uses specific Zotero folders (categorized into stated theoretical and/or methodological approaches) that they are responsible for analyzing. We decided to separate the articles this way to make use of the various theoretical and methodological expertise on our research team.

### Data synthesis

Our findings will be synthesized in quantitative and qualitative forms. The results will be presented in the form of a chart that will list the different lenses that are used in Indigenous women’s health and wellness research, how often they are used, which topics of research they are used for, and in which disciplines. Once this data has been collated, we will share these results with our broader community of Indigenous colleagues who work in the field of Indigenous health research to identify strengths and gaps informing the anti-oppressive research tool. For our knowledge sharing plan, we aim to begin the discussions of our scoping review results with our Indigenous scholars and community members that we have long-standing relationships with. Alongside these colleagues and peers, we will determine the next steps of our research creation contributing to the field of Indigenous women’s health and wellness.

## Discussion

Research on Indigenous women’s health and wellness in North America continues to ignore and/or dismiss the contributions of Indigenous peoples’ perspectives, knowledge, methods, epistemologies, and methodologies [3]. Specific to Indigenous women involved in Indigenous health research, Anderson and Cidro’s [2] findings link to the systemic complexities entangled with gendered experience of community-engaged research. They and other scholars [30] point to the implications and future directions to address the structural inequities involved in Indigenous health research and to be grounded in Indigenous women’s perspectives. A gendered view is needed to address gendered-violence and “to build a decolonial feminist resistance” [31]. The use of Indigenous research methodologies carries the potential to address imbalanced/gendered violence/deficit-based health research as it is informed by an Indigenous worldview with its ethics of kinship relationality [2, 3, 8, 15, 20, 32–38]. In doing so, we increasingly recognize that Indigenous women were traditionally and continue to be the community health researchers and are well equipped with tools, skills, land knowledge, traditional laws, languages, and kinship value systems [39–42]. Building on Indigenous women’s vision to encourage and support decolonial health research, we aim to contribute to Indigenous women’s health scholarship by pointing to theoretical frameworks’ that risk upholding gendered violence and white supremacy by rendering invisible the ongoing intersectional oppressions of patriarchy and settler-colonialism [13, 31].

The results of our scoping analysis will map out the current field of Indigenous women’s health research. We will do this by identifying, categorizing, and analyzing research conducted from CBPR and critical theoretical lenses over the past decade. As far as we are aware, there has not been another scoping review completed on this nascent topic. Over the next few months, we will continue to extract and analyze data from our selected articles. Our aim for the analysis will be to demonstrate the disciplines engaged in Indigenous women’s health and the ways in which theoretical lenses shape the research questions, methodologies, analysis, and implications of the research. This will provide a clear picture of what is missing and where the field needs to grow. In order to stay on top of the most recent work in the field, our scoping analysis methodology will allow us to continually circle back to take newer articles through the screening process and include them in our analysis.

Once we have this data, we will expand our circle to include more decolonial scholars in Indigenous women’s health and well-being to co-construct an anti-oppressive tool intended to guide researchers through the various theoretical lenses that result in research that is reflective, respectful, and reciprocal.

Potential limitations we anticipate for this study are that we may have missed more recent article, those conducted in languages other than English, and those using terms other than “gender” to classify their research. For example, our search terms were in English but increasingly, Indigenous researchers are using Indigenous languages in their work. As well, two-spirited health research is emerging. By sharing our results with our community of Indigenous health researchers and asking for feedback on missing works, we hope to mitigate some of these limitations.

## Data Availability

The datasets used and/or analyzed during the current study are available from the corresponding author on reasonable request.
